# Impaired regulatory function of granzyme B-producing B cells against T cell inflammatory responses in lupus mice

**DOI:** 10.1136/lupus-2023-000974

**Published:** 2023-07-27

**Authors:** Jimeng Xue, Liling Xu, Hua Zhong, Mingxin Bai, Xin Li, Ranran Yao, Ziye Wang, Zhen Zhao, Hongchao Li, Huaqun Zhu, Fanlei Hu, Yin Su

**Affiliations:** 1 Department of Rheumatology and Immunology, Peking University People’s Hospital & Beijing Key Laboratory for Rheumatism Mechanism and Immune Diagnosis (BZ0135), Peking University People’s Hospital, Beijing, China; 2 Peking-Tsinghua Center for Life Sciences, Peking University, Beijing, China; 3 Department of Rheumatology and Immunology, Beijing Jishuitan Hospital, Beijing, China; 4 State Key Laboratory of Natural and Biomimetic Drugs, School of Pharmaceutical Sciences, Peking University, Beijing, China; 5 Department of Integration of Chinese and Western Medicine, School of Basic Medical Sciences, Peking University, Beijing, China

**Keywords:** B cells, T cells, autoimmunity, lupus erythematosus, systemic, B-lymphocytes

## Abstract

**Objective:**

Recently, a new subtype of granzyme B (GrB)-producing Breg cells has been identified, which was proven to be involved in autoimmune disease. Our recent report demonstrated that GrB-producing Breg cells were correlated with clinical and immunological features of SLE. However, the effect of GrB-producing Breg cells in lupus mice is unclear.

**Methods:**

GrB expression in naïve and lupus mouse B cells was analysed using flow cytometry, PCR, ELISA and ELISpot assays. To study the role of GrB-producing B cells in a lupus model, GrB knockout (KO) and wild-type (WT) mice were intraperitoneally injected with monoclonal cells from the mutant mouse strain B6.C-H-2bm12 (bm12) for 2 weeks. In addition, the function of GrB-producing Breg cells in naïve and lupus mice was further explored using in vitro B cells-CD4^+^CD25^−^ T cell co-culture assays with GrB blockade/KO of B cells.

**Results:**

B cells from the spleens of WT C57BL/6 (B6) mice could express and secret GrB (p<0.001). GrB-producing Breg cells from WT mice showed their regulatory functions on CD4^+^CD25^−^ T cell. While the frequency of GrB-producing Breg cells was significantly decreased (p=0.001) in lupus mice (p<0.001). Moreover, GrB-producing Breg cells in lupus mice failed to suppress T cell-mediated proinflammatory responses, partially due to the impaired capacity of downregulating the T cell receptor-zeta chain and inducing CD4^+^CD25^−^ T cell apoptosis.

**Conclusion:**

This study further revealed the function and mechanism of GrB-producing Breg cells in regulating T cell homeostasis in lupus mice and highlighted GrB-producing Breg cells as a therapeutic target in SLE.

**WHAT IS ALREADY KNOWN ON THIS TOPIC**

- Dysregulated B cells play a crucial role in the development of SLE.
- Recently, a unique Breg cell subset that produce granzyme B (GrB) has been identified in humans and reported to be actively involved in the pathogenesis of SLE; however, a full understanding of the role of GrB-producing Breg cells in mouse models of lupus remains elusive.

**WHAT THIS STUDY ADDS**

- This study confirmed the existence of GrB-producing Breg cells and revealed their regulatory functions in mice.
- These cells were abnormally decreased in lupus mice and failed to exert potent immunosuppressive effects in bm12-induced lupus mice.

**HOW THIS STUDY MIGHT AFFECT RESEARCH, PRACTICE OR POLICY**

- As GrB-producing Breg cells possess important regulatory properties against CD4^+^CD25^−^ T cells, targeting them may provide new approaches to improve SLE therapy.

## Background

SLE is a multisystem autoimmune disease characterised by T cell and B cell dysfunction, production of multiple autoantibodies and end-organ damage.^1 2^ Although the aetiology and pathogenesis of SLE remain unclear, the abnormalities in B cells appear to play pivotal roles in the pathogenesis of SLE.^3 4^

B cell dysregulation plays a crucial role in SLE because of the ability of B cells to produce autoantibodies, secrete proinflammatory cytokines and present antigens to regulate immune responses.^5 6^ In healthy individuals, with the capacity to induce T cell activation and trigger humoral responses, B cells are generally considered effectors of the immune system.^7^ The recent discovery of regulatory B cells (Bregs) has provided new insights into the role of B cells in immune responses beyond antibody production.^8^ Various types of human and murine Bregs have been reported to suppress inflammatory responses in infections, autoimmune diseases, allergies, transplantation and cancer.^9–12^ The key suppressive molecules that exert their regulatory functions include the cytokines interleukin (IL)-10, transforming growth factor (TGF)-β and IL-35, as well as cell membrane-bound molecules, such as the aryl hydrocarbon receptor, programmed death-ligand 1, CD73 and CD39.^13 14^ The phenotypic features of human Bregs have traditionally been identified as a CD19^+^CD24^high^CD38^high^ B cell population;^15^ however, a unified phenotypic definition is still lacking. Bregs are characterised by the release of IL-10, and the capacity of CD19^+^ B cells to release IL-10 is significantly reduced in patients with SLE, especially in those with lupus nephritis.^16^

Recently, a newly discovered B cell subset secreting granzyme B (GrB) was identified as suppressive B cells, which are involved in several diseases, like infections,^17^ B cell chronic lymphocytic leukemia,^18^ autoimmune diseases,^19 20^ renal and liver dysfunction^21 22^ and cancer.^23 24^ GrB is a member of the serine protease family and is traditionally known to be produced by natural killer cells (NKs) and cytotoxic T cells (CTLs) to induce apoptosis in target cells.^25^ However, an increasing number of studies have demonstrated that the production of GrB by B cells is not accompanied by perforin, as is the case with many other GrB-producing cells, suggesting that GrB may have extracellular activity.^26 27^ In 2006, Jahrsdörfer *et al*^18^ first discovered that B cells were capable of producing GrB and acquiring cytotoxic potential in the presence of IL-21. Since then, several studies have shown that GrB expressed by regulatory T cells (Tregs) or plasmacytoid dendritic cells (pDCs) can mediate perforin-independent degradation of the T cell receptor (TCR)-zeta chain, resulting in immunosuppressive effects on effector T cells.^28–30^ Lindner *et al*^23^ reported that GrB-producing Breg cells limit the proliferation of T cells by promoting the degradation of the TCR-zeta chain in a perforin-independent manner. Following these studies, our group observed that GrB-producing Breg cells were capable of inhibiting CD4^+^ T cell proliferation through mechanisms involving GrB-mediated TCR-zeta chain degradation and T cell apoptosis.^19 20^

In addition, GrB-producing Breg cells have been reported to increase in individuals vaccinated against viral diseases, implying an immunoregulatory potential in antiviral immune responses.^31^ Numerous studies have proposed a role for GrB-producing Breg cells in controlling inflammatory processes because alterations in these B cell subsets have been described in immune-related diseases. For instance, patients requiring renal transplantation show a diminished fraction of GrB-producing Breg cells, which may play a dual role in maintaining allospecific tolerance and enhancing viral control.^21 32^ GrB-producing Breg cells can also prevent effector T cell proliferation in rejection-tolerant patients.^33^ Zhu *et al*^34^ showed that the combination of interferon (IFN)-γ^+^CD4^+^ T cells and GrB^+^CD19^+^ B cells could be used as a potential prognostic marker for acute rejection in liver transplant recipients. Likewise, functional impairments in GrB-producing Breg cells have also been found in patients with rheumatoid arthritis (RA) and SLE, which can be reversed after clinical remission.^19 20^ Rabani *et al*^35^ also found a reduced proportion of GrB-producing Breg cells in patients with SLE, which may promote the development of lupus nephritis. Nevertheless, the exact pathogenesis and potential role of GrB-producing Breg cells in lupus mice remain to be elucidated.

In the present study, we confirmed the presence of GrB-producing Breg cells and revealed their regulatory functions in mice. GrB-producing Breg cells are insufficient and functionally impaired in lupus mice, and provide new insights into the role of B cells in lupus pathogenesis.

## Materials and methods

### Laboratory mice

Old female C57BL/6J mice (B6) aged 6–8 weeks, GrB knockout (KO) mice with a B6 background and male I-A^bm12^B6(C)-H2-Ab1^bm12^/KhEgJ (bm12) mice aged 8–10 weeks were obtained from Jackson Laboratory (Bar Harbor, Maine, USA). All mice were bred and housed in a specific pathogen-free (SPF) environment under controlled conditions (12 hours light/dark cycle, 22°C ambient temperature, 40% humidity).

### Animal model

The lupus mouse model was established as described elsewhere.^36^ Briefly, splenocytes from bm12 mice were obtained as a single cell suspension and then injected intraperitoneally to B6 mice with 200 μL splenocytes per mouse on day 0. Serum was collected on day 14, the mice were euthanised and samples were collected for further analyses.

### Antibodies and reagents

Mouse GrB Antibody (Cat# AF1865), Normal Goat IgG Control (Cat# AB-108-C) and the Mouse GrB ELISpot Development Module (Cat# SEL1865) were purchased from R&D Systems (Minneapolis, Minnesota, USA). Mouse ANA IgG Antibody Assay Kit (Cat# FA1510-1) was purchased from Oumeng (Beijing, China). The Mouse Anti-double-stranded DNA (anti-dsDNA) IgG Antibody Assay Kit (Cat# 3031) was purchased from Chondrex (Redmond, Washington, USA). Recombinant Murine IL-21 (Cat# 210-21-10) was purchased from PerproTech (Rocky Hill, Connecticut, USA). F(ab′)2-goat antimouse IgM (Cat# 16-5092-85), PE antimouse GrB (Cat# 61-8898-82), PerCP/Cyanine5.5 antimouse CD3ε (Cat# 45-0031-82) were purchased from eBioscience (San Diego, California, USA). purified antimouse CD3ε (Cat# 100340), purified antimouse CD28 (Cat# 102116), APC/Fire 750 antimouse CD4 (Cat# 100460), PerCP/Cyanine5.5 antimouse CD19 (Cat# 115534), FITC antimouse CD19 (Cat# 115506), Pacific Blue antimouse CD49b (Cat# 108918), PE/Cy7 antimouse CD25 (Cat# 101916), PerCP/Cyanine5.5 antimouse IFN-γ (Cat# 505822), PE antimouse IL-17A (Cat# 506904), APC Annexin V Apoptosis Detection Kit with 7-aminoactinomycin D (7-AAD) (Cat# 640930) were purchased from BioLegend (San Diego, California, USA). Phorbol 12-myristate 13-acetate (PMA), brefeldin A (BFA) and ionomycin were purchased from Multisciences (Hangzhou, China). RPMI 1640 medium, 1% penicillin/streptomycin and fetal bovine serum were purchased from Invitrogen (Carlsbad, California, USA).

### Determination of ANA and double-stranded DNA antibodies

Serum was obtained from lupus and naïve mice after 2 weeks, as described above. The concentrations of ANA IgG and anti-dsDNA antibodies were measured using the corresponding ELISA kits according to the manufacturer’s protocols.

### Flow cytometry

For intracellular staining, cells were incubated with PMA (50 ng/mL), BFA (10 μg/mL) and ionomycin (1 μg/mL) for 5 hours, and then surface-stained, fixed, permeabilised and intracellularly stained according to the manufacturer’s instructions. For the expression of GrB on CD19^+^ B cells, cells were stained with anti-CD19, anti-CD3ε, anti-CD49b. Apoptotic cells were detected by staining with 7-AAD and APC Annexin V according to the manufacturer’s instructions. The corresponding negative/positive isotype staining was performed. The cells were then analysed using FACS Arial II, and the results were further analysed using FlowJo 10 data analysis software.

CD19^+^ B, CD4^+^CD25^−^ T and CD8a^+^ T cells were isolated from mouse spleen single-cell suspensions, and the gating strategies are presented in online supplemental figure S1. The purity used for the experiments was 95%–99%. The gating strategies for the T helper (Th)1, Th2 and TCR-zeta chains are presented in online supplemental figure S2.

10.1136/lupus-2023-000974.supp1 Supplementary data

10.1136/lupus-2023-000974.supp2 Supplementary data

### qPCR analysis of GrB expression

The expression of GrB mRNA in CD19^+^ B cells from lupus mice and controls was analysed using real-time quantitative PCR (qPCR) according to the manufacturer’s instructions. The sequences of the primers used in this study were as follows: the forward β-actin primer was 5′-CCACTCTCGACCCTACATGG-3′, the reverse β-actin primer was 5′- GGCCCCCAAAGTGACATTTATT-3′, the forward GrB primer was 5′-TGACAGGATGCAGAAGGAGA-3′, the reverse GrB primer was 5′-GAGGCATGCCATTGTTTCGTC-3′.

### ELISpot assay

GrB ELISpot assays were performed according to the manufacturer’s protocol.

### In vitro cell culture

CD19^+^ B cells and CD4^+^CD25^−^ T cell spleens of mice were isolated using flow cytometry. The purity used in the experiments was 95%–99%. Then 5×10^6^ CD19^+^ B cells were cocultured with 1×10^6^ CD4^+^CD25^−^ T cells (5:1) in the presence of plate-bound anti-CD3ε (1 μg/mL), and with anti-CD28 (1 μg/mL), anti-GrB antibody (5 µg/mL) or isotype antibody (5 µg/mL), IL-21 (50 ng/mL), CPG (10 μg/mL), IgM (10 μg/mL) for 72 hours. Cells were harvested for flow cytometry as previously described.

### Statistical analysis

Statistical analysis was performed using the statistical software program SPSS V.24.0 (SPSS, Chicago, Illinois, USA). Differences between various groups were evaluated using Student’s t-test and the non-parametric Mann-Whitney U test. P value <0.05 was considered statistically significant (*p<0.05, **p<0.01, ***p<0.001, and not significant (ns)).

## Results

### Production of GrB by B cells in mice spleen

**Figure 1:**
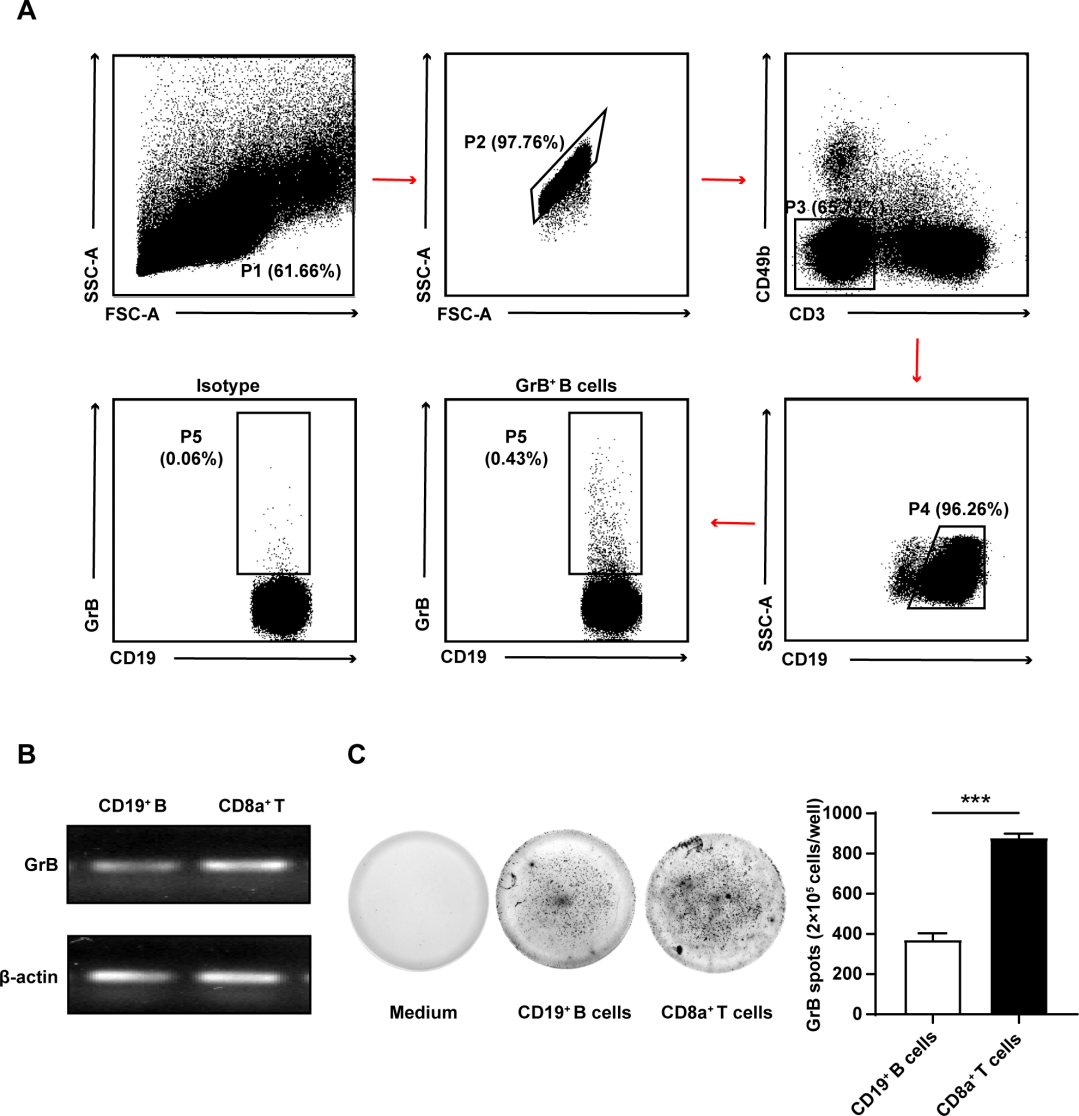
B cells from the mice spleen produced granzyme B (GrB) spontaneously. (A) Spleen single-cell suspensions were isolated from B6 mice and incubated with brefeldin A (BFA) (10 µg/mL), ionomycin (1 µg/mL) and phorbol 12-myristate 13-acetate (PMA) (50 ng/mL) for 5 hours. The expression of GrB in CD19^+^ B cells was detected by staining with anti-CD19, anti-CD3ε, anti-CD49b and anti-GrB. FACS gating strategy for identifying the expression of GrB on CD19^+^ B cells was shown. (B) Flow cytometry-sorted CD19^+^ B cells (1×10^6^) from the spleen of B6 mice were set to detect the mRNA expression of GrB by PCR. (C) Freshly purified CD19^+^ B cells (2 × 10^5^; middle) from B6 spleen were cultured with CpG (10 µg/mL) stimulation on mice GrB-specific ELISpot plates for 24 hours. Medium (left) and CD8a^+^ T cells (right) were used as blank control and positive control, respectively. Dots were counted and the representative of independent data from five different B6 mice was shown (p<0.001). ***p<0.001 (Student’s t-test C).

First, to determine whether spleen B cells of B6 mice could produce GrB, single-cell suspensions were isolated from the spleens of B6 mice (n=10) for further staining with anti-CD19, anti-CD3, anti-CD49b and anti-GrB and then detected by flow cytometry. The results showed the spleen B cells of B6 mice (CD3^−^CD49b^−^CD19^+^) were able to produce GrB (figure 1A). To confirm our findings, we verified the mRNA expression of GrB in purified B cells using PCR (figure 1B). We then performed a GrB-specific ELISpot analysis to investigate whether B cells secrete GrB. Compared with CD8a^+^ T cells, CD19^+^ B cells in B6 mice also secreted GrB (p<0.001) (figure 1C).

### Mouse GrB-producing Breg cells exerted immunosuppressive functions against CD4^+^CD25^−^ effector T cells

**Figure 2:**
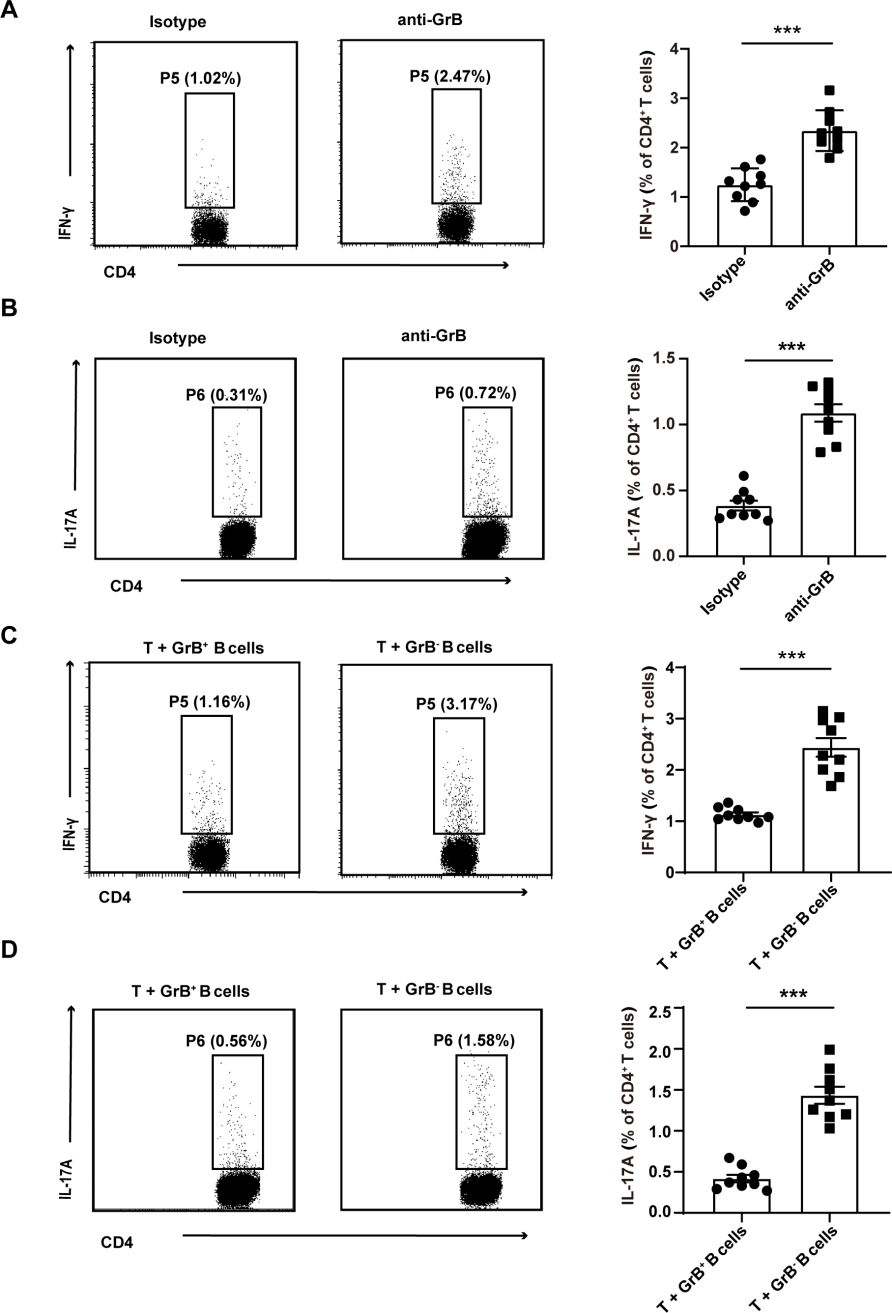
Granzyme B (GrB)-producing B cells demonstrated immunosuppressive functions against CD4^+^ T cells in mice. Spleen CD19^+^ B cells (5×10^6^) and CD4^+^CD25^−^ T cells (1×10^6^) from B6 mice (n=9) were cocultured with anti-CD3ε (1 μg/mL, 4°C overnight), anti-CD28 (1 μg/mL), CPG (10 μg/mL), IgM (1 μg/mL), recombinant human interleukin (IL)-21 (rhIL-21, 50 ng/mL) in the presence of anti-GrB (5 μg/mL) or isotype control (5 μg/mL) for 72 hours. The proportion of T helper (Th)1 (p<0.001) (A) and Th17 cells (p<0.001) (B) increases significantly in the presence of anti-GrB. CD19^+^ B cells (5×10^6^) from B6 mice (GrB^+^ regulatory B cells (Bregs)) or GrB knockout (KO) mice (GrB^-^ Breg) and CD4^+^CD25^−^ T cells (1×10^6^) from B6 mice (n=9) were cocultured with anti-CD3ε (1 μg/mL, 4°C overnight), anti-CD28 (1 μg/mL), CPG (10 μg/mL), IgM (1 μg/mL), rhIL-21 (50 ng/mL) for 72 hours. The frequencies of Th1 (p<0.001) (C) and Th17 (p<0.001) (D) were significantly higher in the presence of GrB^-^ Breg cells than GrB^+^ Breg cells. ***p<0.001 (Student’s t-test A–D). IFN, interferon.

To functionally evaluate the role of GrB-producing B cells, CD4^+^CD25^−^ T cells and CD19^+^ B cells from the spleens of B6 mice were cocultured for 72 hours with anti-GrB antibody or isotype control antibody, and the frequencies of Th1 and Th17 cells were detected by flow cytometry. The proportion of Th1 and Th17 cells was significantly elevated in the presence of anti-GrB antibody (p<0.001, p<0.001) (figure 2A,B).

Then, GrB KO mice were used to further verify the results, CD19^+^ B cells from the spleen of B6 mice (GrB^+^ B) or GrB KO mice (GrB^−^ B) were cocultured with CD4^+^CD25^−^ T cells from B6 mice for 72 hours, the frequencies of Th1 and Th17 cells were detected by flow cytometry. The results also demonstrated that the frequencies of Th1 and Th17 cells were significantly increased in the presence of GrB^+^ B cells (p<0.001, p<0.001) (figure 2C,D). These results indicated that GrB-producing B cells possess potential negative immunoregulatory functions and that the new B cell subset may be termed GrB-producing Breg cells.

### Mouse GrB-producing Breg cells induced CD4^+^CD25^−^ T cell apoptosis and promoted TCR-zeta chain degradation

**Figure 3:**
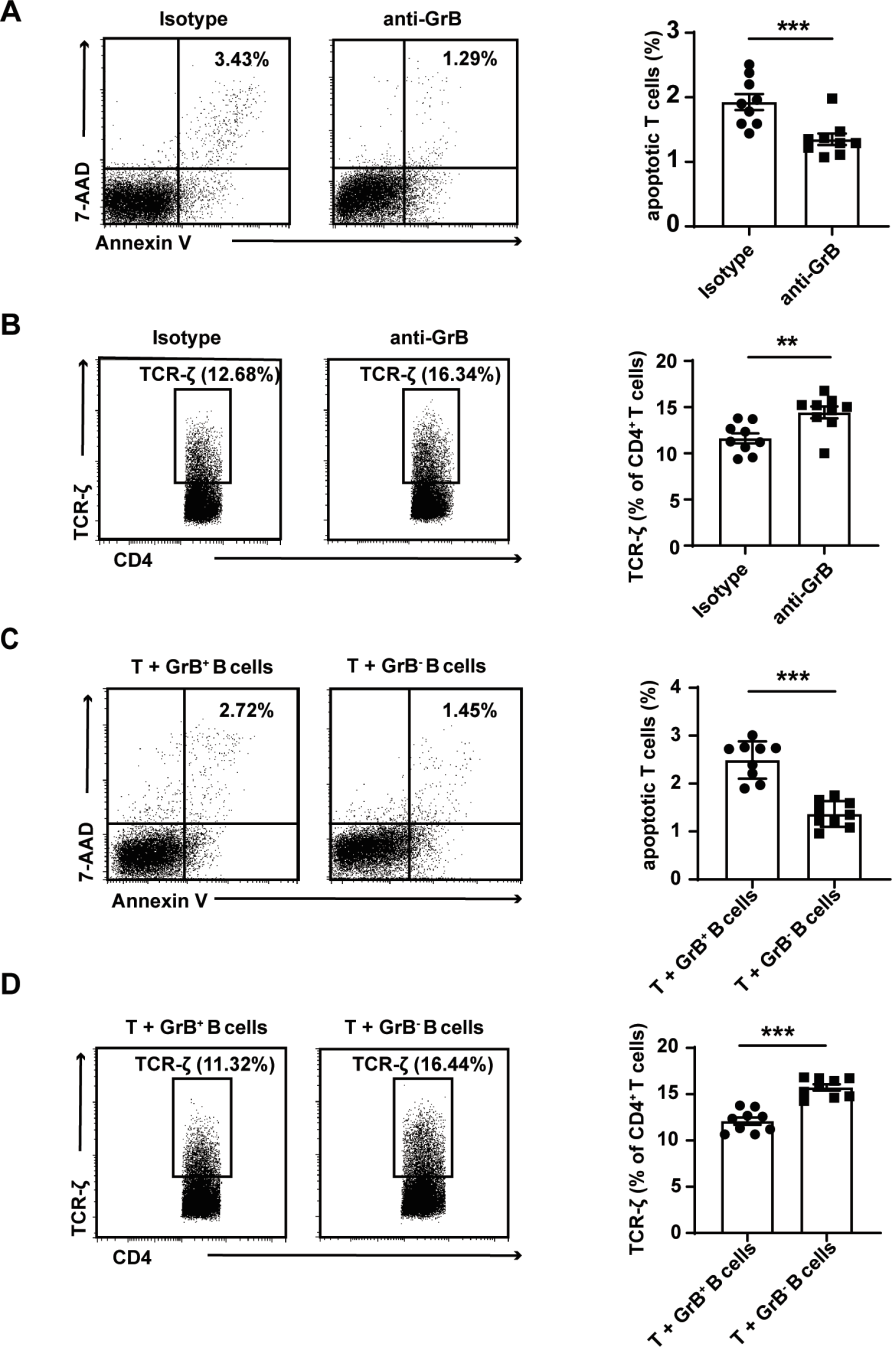
Granzyme B (GrB)-producing Breg cells induced CD4^+^ T cell apoptosis and downregulated the expression of the T cell receptor (TCR)-zeta chain. Spleen CD19^+^ B cells (5×10^6^) and CD4^+^CD25^−^ T cells (1×10^6^) from B6 mice (n=9) were cocultured in the presence of anti-GrB antibody (5 μg/mL) or isotype control (5 μg/mL) for 72 hours. The frequencies of apoptotic (7-aminoactinomycin D (7-AAD)^+^Annexin V^+^) T cells (p<0.001) (A) and TCR-zeta^+^ T cells (p=0.004) (B) were detected by flow cytometry and the representative dots (left) as well as the statistical results (right) were shown in the presence of anti-GrB. CD19^+^ B cells (5×10^6^) from B6 (GrB^+^ regulatory B cells (Bregs)) or GrB knockout (KO) mice (GrB^-^ Breg) and CD4^+^CD25^−^ T cells (1×10^6^) from B6 mice (n=9) were cocultured for 72 hours. The frequencies of apoptotic T cells (p<0.001) (C) and TCR-zeta^+^ T cells (p<0.001) (D) were detected by flow cytometry and the representative dots (left) as well as the statistical results (right) were shown in the presence of GrB^-^ Breg cells than GrB^+^ Breg cells. **p<0.01, ***p<0.001 (Student’s t-test A–D).

To further explore the possible regulatory role of GrB-producing Breg cells, CD4^+^CD25^−^ T cells and CD19^+^ B cells from the spleens of B6 mice were cocultured for 72 hours in the presence of anti-GrB antibody or isotype control antibody, and the expression of apoptotic T cells and TCR-zeta^+^ T cells was detected by flow cytometry. We found that the frequency of apoptotic T cells was significantly decreased when GrB was blocked (p<0.001) (figure 3A). Moreover, the TCR-zeta chain expression was upregulated when GrB was neutralised (p=0.004) (figure 3B).

To further verify the above results, B cells isolated from the spleens of B6 mice (GrB^+^ B cells) or GrB KO mice (GrB^−^ B cells) were separately cocultured with CD4^+^CD25^−^ T cells from the spleens of B6 mice for 72 hours, and the frequencies of apoptotic T cells and TCR-zeta^+^ T cells were detected by flow cytometry. The percentage of apoptotic T cells was significantly increased in the presence of GrB^+^ Breg cells (p<0.001) (figure 3C). TCR-zeta chain expression was downregulated in the presence of GrB^+^ B cells (p<0.001) (figure 3D). These results indicated that GrB-producing Breg cells play an immunomodulatory role by promoting T cell apoptosis and TCR-zeta chain degradation.

### Reduced frequencies of GrB-producing Breg cells in lupus mice

**Figure 4:**
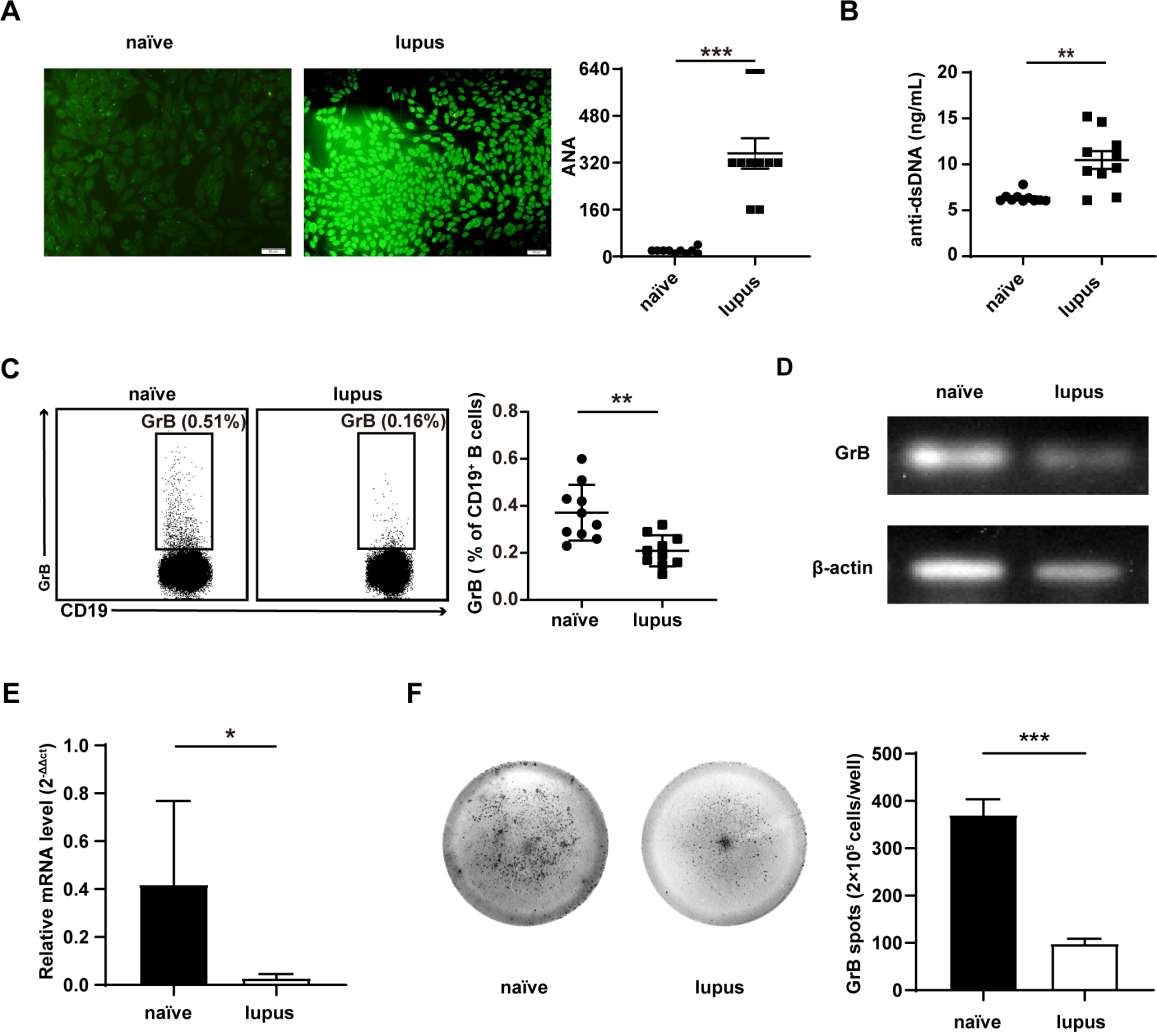
Reduced granzyme B (GrB)-producing Breg cells in lupus mice. Bm12 mice spleen lymphocytes (1.2×10^8^ cells) were injected intravenously into indicated animals (aged 6–8 weeks). Representative anti-ANAs (p<0.001) (A) staining and ELISA analysis of anti-double-stranded DNA (anti-dsDNA) (p=0.002) (B) of serum from mice described in A–B at 14 days. (C) The frequencies of GrB-producing Breg cells were assayed by flow cytometry in lupus (n=10), and naïve mice (n=10), the representative dots (left) and statistical results were shown (right) (p=0.001). Purified CD19^+^ B cells from lupus (n=5) and naïve mice (n=5) were subjected to detection of mRNA expression of GrB by PCR (left) (D) and quantitative PCR (right) (p=0.037) (E). CD19^+^ B cells (2.5×10^5^ cells/well) from lupus (n=5) and naïve mice (n=5) were cultured with CpG stimulation (10 µg/mL) on specific mice GrB ELISpot plates for 24 hours. The representative figures (left) and statistical results (right) were shown (p<0.001) (F). *p<0.05, **p<0.01, ***p<0.001 (Student’s t-test C, E, F and Mann-Whitney U test A, B).

To examine the production of GrB-producing Breg cells in lupus mice, wild-type mice were intraperitoneally injected with monoclonal cells from the bm12 mice. Representative anti-ANAs staining and ELISA analysis of anti-dsDNA in the serum of mice are shown in figure 4A,B. The production of anti-ANAs (p<0.001) (figure 4A) and anti-dsDNA IgG (p=0.002) (figure 4B) were markedly increased in bm12-induced lupus mice. To determine the expression of GrB-producing Breg cells in lupus mice, the frequencies of GrB-producing Breg cells in lupus (n=10) and naïve mice (n=10) were analysed using flow cytometry. Interestingly, compared with naïve mice, the number of GrB-producing Breg cells was significantly decreased in lupus mice (p=0.001) (figure 4C). Furthermore, PCR, qPCR (p=0.037) and mice-specific ELISpot assays (p<0.001) were performed, and reduced GrB-producing Breg cells in lupus mice were confirmed (figure 4D–F).

### The impaired immunosuppressive capacity of GrB-producing Breg cells in lupus mice

**Figure 5:**
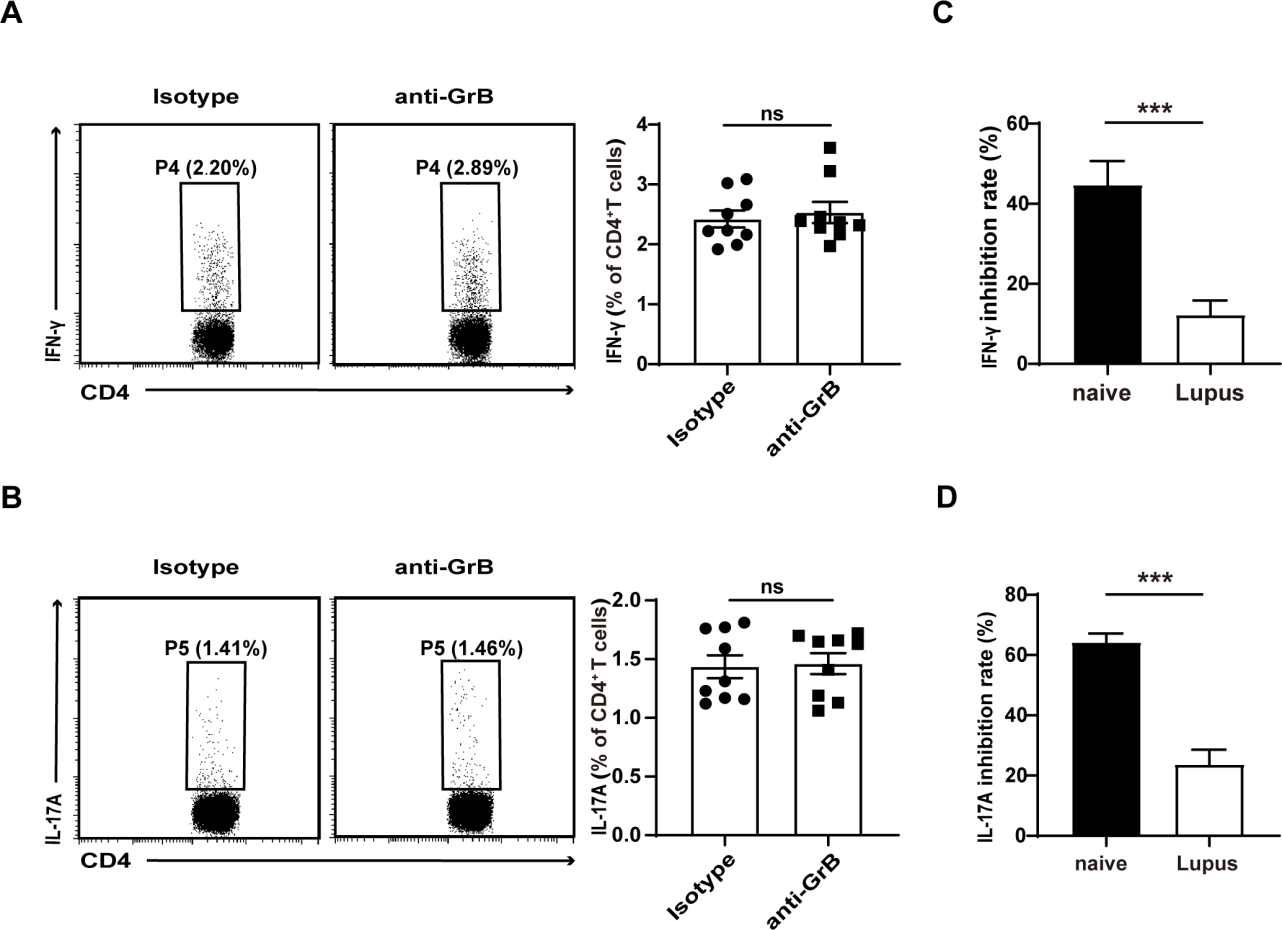
Granzyme B (GrB)-producing regulatory B cells failed to suppress CD4^+^ T cell responses in lupus mice. (A, B) Spleen CD19^+^ B cells and CD4^+^CD25^−^ T cells from lupus mice (n = 9) were purified and cocultured in the presence of the anti-GrB or isotype control. The representative dots of T helper (Th)1 (A) and Th17 (B) cells were measured by flow cytometry, and there was no significant difference between the anti-GrB and isotype control groups. (C, D) The inhibition rates of GrB-producing Breg cells against Th1 (p<0.001) (C) and Th17 (p<0.001) (D) cells both in lupus and naïve mice were calculated using the following formula: inhibition rates=($\frac{Th1/Th17\;frequencies\;on\;anti-GrB\;group-Th1/Th17\;frequencies\;on\;isotype\;group}{Th1/Th17\;frequencies\;on\;anti-GrB\;group}$)×100%. ***p<0.001, ns, not significant (Student’s t-test C, D; Mann-Whitney U test A, B). IFN, interferon; IL, interleukin.

Finally, we analysed the suppressive function of GrB-producing Breg cells in lupus mice. CD19^+^ B cells and CD4^+^CD25^−^ T cells were purified from the spleens of lupus mice and cocultured with or without GrB blockade. The results showed no significant effects on the expression of Th1 and Th17 cells (figure 5A,B). Furthermore, the inhibition rates of GrB-producing Breg cells in both lupus and naïve mice further confirmed their impaired suppressive functions (p<0.001, 0.001) (figure 5C,D). These results indicated an impaired suppressive capacity of GrB-producing Breg cells in lupus mice.

## Discussion

In this study, we further demonstrated that mouse B cells could produce GrB, and these GrB-producing Breg cells negatively regulate Th1 and Th17 cell responses by inducing T cell apoptosis and downregulating the TCR-zeta chain. In lupus mice, GrB-producing Breg cells are significantly reduced and functionally impaired.

GrB is a member of the serine protease family which mediates target cell apoptosis via perforin-mediated membrane rupture after entry into the cytoplasm.^37^ Although GrB has been widely described as a part of the cytotoxic machinery of activated NT cells and CTL, Jahrsdörfer *et al*^18^ demonstrated that human B cells can secrete GrB and possess cytotoxic potential. Hagn *et al*^38^ found that peripheral blood B cells from RA, SLE, psoriasis, healthy individuals and cord blood could express and secrete GrB. GrB-producing Breg cells are significantly increased in patients with primary Sjögren’s syndrome and inflammatory bowel disease and are negatively correlated with disease activity.^39 40^ Our previous study also found functional impairments in GrB-producing Breg cells in patients with RA and SLE, which could be reversed after clinical remission.^19 20^ This study showed that GrB-producing Breg cells displayed potent regulatory properties and impaired GrB-producing Breg cells were present in lupus mice. However, no significant difference was noted in the incidence of lupus between the B6 and GrB KO mice (data not shown). Considering that GrB is produced in a complex environment, it plays a cytotoxic role on CTL and NK cells. However, the detailed mechanisms of GrB-producing Breg cells need to be studied further.

B cell subsets with immunosuppressive properties play critical roles in infection, inflammation, autoimmunity, transplantation and physiological conditions.^10 12 15^ Several studies have shown that different Breg subsets exert their inhibitory effects by secreting corresponding cytokines, such as IL-10, IL-35 and TGF-β.^14^ In addition to the broadly described Bregs, GrB-producing Breg cells are new Breg subsets with potent regulatory properties.^41^ Other regulatory cell populations that express GrB, such as Tregs and pDCs, exert immunosuppressive functions possibly mediated by a perforin-independent pathway by inducing TCR-zeta chain degradation.^28 30^ Following these studies, Lindner *et al*^23^ demonstrated that GrB-producing Breg cells may also limit T cell proliferation by inducing degradation of the TCR-zeta chain. Our previous studies suggest that GrB-producing Breg cells can inhibit CD4^+^ T cell proliferation through mechanisms involving GrB-mediated TCR-zeta chain degradation and T cell apoptosis,^19 20^ which was also clarified in B6 mice in this study.

To understand the role of GrB-producing Breg cells, we measured their expression in mice. Previously, Hagn *et al*^42^ found that the expression of GrB in C57BL/6, BALB/c, DBA/2 and CBA mouse B cells was lacking, whereas our results suggest that B6 mice could express small amounts of GrB-producing Breg cells. Although the proportion of these B cell subsets is low, GrB-producing Breg cells may also play a pivotal role in mice, given the overwhelming proportion of B cells in the spleens of B6 mice.

Unfortunately, only intracellular staining can be used to identify GrB-producing Breg cells using flow cytometry and there are no reliable surface markers, limiting the study of this B cell subset. In 2003, Lindner *et al*^23^ firstly identified cell surface biomarkers of GrB-producing Breg cells as CD19^+^CD38^+^CD1d^+^IgM^+^CD147^+^. Several studies have proposed that cell surface markers may also include CD19^+^IgG^+^IgD^−^CD27^−^,^43^ CD38^+^CD20^−^,^44^ CD5^+^CD38^+^CD27^+^CD138^+^CD19^+^^45^ or CD138^+^CD27^+^CD5^+^CD38^+^IgD^−^.^32^ Nevertheless, the unique surface markers of GrB-producing Breg cells remain controversial, and further studies are required to identify these surface markers.

Furthermore, our previous studies have shown reduced expression of GrB-producing Breg cells in patients with SLE and RA,^19 20^ and we had found a decreased proportion of GrB-producing Breg cells in lupus mice compared with that in naïve mice. This study demonstrated that GrB-producing Breg cells can negatively regulate CD4^+^ T cell proliferation, partly by inducing T cell apoptosis and downregulating the TCR-zeta chain. Thus, it is intriguing to hypothesise that GrB-producing Breg cells have an apparent inhibitory effect on T cell proliferation in SLE. Nevertheless, the detailed signalling mechanisms of GrB-producing Breg cells in SLE require further elucidation.

In summary, this is the first study to demonstrate that mouse B cells express GrB and exert immunosuppressive functions. The results revealed that the frequency of GrB-producing Breg cells decreased dramatically in lupus mice. Notably, these cells could inhibit CD4^+^CD25^−^ T cell inflammatory responses by reducing TCR-zeta chain expression and inducing T cell apoptosis, whereas the regulatory function of these cells on T cells is impaired in lupus mice. Thus, we demonstrated that the impairment of GrB-producing Breg cells may be related to the pathogenesis of lupus in mice and provide a new therapeutic target for the treatment of SLE. However, the detailed mechanisms of these cells need to be further studied.

## Data Availability

Data are available on reasonable request.

## References

[1] Kaul A, Gordon C, Crow MK, et al. Systemic lupus erythematosus. Nat Rev Dis Primers 2016;2:16039. 10.1038/nrdp.2016.39 27306639

[2] Durcan L, O’Dwyer T, Petri M. Management strategies and future directions for systemic lupus erythematosus in adults. Lancet 2019;393:2332–43. 10.1016/S0140-6736(19)30237-5 31180030

[3] Tipton CM, Hom JR, Fucile CF, et al. Understanding B-cell activation and autoantibody repertoire selection in systemic lupus erythematosus: A B-cell Immunomics approach. Immunol Rev 2018;284:120–31. 10.1111/imr.12660 29944759 PMC6022284

[4] Yap DYH, Chan TM. B cell abnormalities in systemic lupus erythematosus and lupus nephritis-role in pathogenesis and effect of immunosuppressive treatments. Int J Mol Sci 2019;20:6231. 10.3390/ijms20246231 31835612 PMC6940927

[5] Dörner T, Jacobi AM, Lipsky PE. B cells in Autoimmunity. Arthritis Res Ther 2009;11:247. 10.1186/ar2780 19849820 PMC2787254

[6] Dörner T, Giesecke C, Lipsky PE. Mechanisms of B cell Autoimmunity in SLE. Arthritis Res Ther 2011;13:243. 10.1186/ar3433 22078750 PMC3308063

[7] Rosser EC, Mauri C. Regulatory B cells: origin, phenotype, and function. Immunity 2015;42:607–12. 10.1016/j.immuni.2015.04.005 25902480

[8] Zhu Q, Rui K, Wang S, et al. Advances of regulatory B cells in autoimmune diseases. Front Immunol 2021;12:592914. 10.3389/fimmu.2021.592914 33936028 PMC8082147

[9] Michaud D, Steward CR, Mirlekar B, et al. Regulatory B cells in cancer. Immunol Rev 2021;299:74–92. 10.1111/imr.12939 33368346 PMC7965344

[10] Alhabbab RY, Nova-Lamperti E, Aravena O, et al. Regulatory B cells: development, phenotypes, functions, and role in transplantation. Immunol Rev 2019;292:164–79. 10.1111/imr.12800 31559645

[11] Ma S, Satitsuksanoa P, Jansen K, et al. B regulatory cells in allergy. Immunol Rev 2021;299:10–30. 10.1111/imr.12937 33345311

[12] Dasgupta S, Dasgupta S, Bandyopadhyay M. Regulatory B cells in infection, inflammation, and Autoimmunity. Cell Immunol 2020;352:104076. 10.1016/j.cellimm.2020.104076 32143836

[13] Jansen K, Cevhertas L, Ma S, et al. Regulatory B cells, A to Z. Allergy 2021;76:2699–715. 10.1111/all.14763 33544905

[14] Catalán D, Mansilla MA, Ferrier A, et al. Immunosuppressive mechanisms of regulatory B cells. Front Immunol 2021;12:611795. 10.3389/fimmu.2021.611795 33995344 PMC8118522

[15] Blair PA, Noreña LY, Flores-Borja F, et al. Cd19(+)Cd24(Hi)Cd38(Hi) B cells exhibit regulatory capacity in healthy individuals but are functionally impaired in systemic lupus erythematosus patients. Immunity 2010;32:129–40. 10.1016/j.immuni.2009.11.009 20079667

[16] Heinemann K, Wilde B, Hoerning A, et al. Decreased IL-10(+) regulatory B cells (Bregs) in lupus nephritis patients. Scand J Rheumatol 2016;45:312–6. 10.3109/03009742.2015.1126346 26948375

[17] Kaltenmeier C, Gawanbacht A, Beyer T, et al. Cd4+ T cell-derived IL-21 and deprivation of Cd40 signaling favor the in vivo development of Granzyme B-expressing regulatory B cells in HIV patients. J Immunol 2015;194:3768–77. 10.4049/jimmunol.1402568 25780036

[18] Jahrsdörfer B, Blackwell SE, Wooldridge JE, et al. B-chronic lymphocytic leukemia cells and other B cells can produce Granzyme B and gain cytotoxic potential after Interleukin-21-based activation. Blood 2006;108:2712–9. 10.1182/blood-2006-03-014001 16809616 PMC1895576

[19] Bai M, Xu L, Zhu H, et al. Impaired Granzyme B-producing regulatory B cells in systemic lupus erythematosus. Mol Immunol 2021;140:217–24. 10.1016/j.molimm.2021.09.012 34749262

[20] Xu L, Liu X, Liu H, et al. Impairment of Granzyme B-producing regulatory B cells correlates with exacerbated rheumatoid arthritis. Front Immunol 2017;8:768. 10.3389/fimmu.2017.00768 28713386 PMC5491972

[21] Zhu J, Zeng Y, Dolff S, et al. Granzyme B producing B-cells in renal transplant patients. Clin Immunol 2017;184:48–53. 10.1016/j.clim.2017.04.016 28461110

[22] Xu W-L, Wang R-L, Liu Z, et al. Granzyme B-producing B cells function as a feedback loop for T helper cells in liver transplant recipients with acute rejection. Inflammation 2021;44:2270–8. 10.1007/s10753-021-01498-9 34120305

[23] Lindner S, Dahlke K, Sontheimer K, et al. Interleukin 21-induced Granzyme B-expressing B cells infiltrate tumors and regulate T cells. Cancer Res 2013;73:2468–79. 10.1158/0008-5472.CAN-12-3450 23384943

[24] Arabpour M, Rasolmali R, Talei A-R, et al. Granzyme B production by activated B cells derived from breast cancer-draining lymph nodes. Mol Immunol 2019;114:172–8. 10.1016/j.molimm.2019.07.019 31357083

[25] Lord SJ, Rajotte RV, Korbutt GS, et al. Granzyme B: a natural born killer. Immunol Rev 2003;193:31–8. 10.1034/j.1600-065x.2003.00044.x 12752668

[26] Boivin WA, Cooper DM, Hiebert PR, et al. Intracellular versus extracellular Granzyme B in immunity and disease: challenging the dogma. Lab Invest 2009;89:1195–220. 10.1038/labinvest.2009.91 19770840 PMC7102238

[27] Hagn M, Jahrsdörfer B. Why do human B cells Secrete Granzyme B? insights into a novel B-cell differentiation pathway. Oncoimmunology 2012;1:1368–75. 10.4161/onci.22354 23243600 PMC3518509

[28] Wieckowski E, Wang G-Q, Gastman BR, et al. Granzyme B-mediated degradation of T-cell receptor Zeta chain. Cancer Res 2002;62:4884–9. 12208735

[29] Gondek DC, Lu L-F, Quezada SA, et al. Cutting edge: contact-mediated suppression by Cd4+Cd25+ regulatory cells involves a Granzyme B-dependent, Perforin-independent mechanism. J Immunol 2005;174:1783–6. 10.4049/jimmunol.174.4.1783 15699103

[30] Jahrsdörfer B, Vollmer A, Blackwell SE, et al. Granzyme B produced by human Plasmacytoid Dendritic cells suppresses T-cell expansion. Blood 2010;115:1156–65. 10.1182/blood-2009-07-235382 19965634 PMC2920226

[31] Hagn M, Schwesinger E, Ebel V, et al. Human B cells Secrete Granzyme B when recognizing viral antigens in the context of the acute phase cytokine IL-21. J Immunol 2009;183:1838–45. 10.4049/jimmunol.0901066 19592644

[32] Chesneau M, Michel L, Dugast E, et al. Tolerant kidney transplant patients produce B cells with regulatory properties. J Am Soc Nephrol 2015;26:2588–98. 10.1681/ASN.2014040404 25644114 PMC4587683

[33] Le Berre L, Chesneau M, Danger R, et al. Connection of Bank1, tolerance, regulatory B cells, and apoptosis: perspectives of a Reductionist investigation. Front Immunol 2021;12:589786. 10.3389/fimmu.2021.589786 33815360 PMC8015775

[34] Zhu J-Q, Wang J, Li X-L, et al. A combination of the percentages of IFN-Gamma(+)Cd4(+)T cells and Granzyme B(+)Cd19(+)B cells is associated with acute hepatic rejection: a case control study. J Transl Med 2021;19. 10.1186/s12967-021-02855-w PMC8088570 33933100

[35] Rabani M, Wilde B, Hübbers K, et al. IL-21 dependent Granzyme B production of B-cells is decreased in patients with lupus nephritis. Clin Immunol 2018;188:45–51. 10.1016/j.clim.2017.12.005 29274388

[36] Klarquist J, Janssen EM. The Bm12 inducible model of systemic lupus erythematosus (SLE) in C57Bl/6 mice. J Vis Exp 2015:53319. 10.3791/53319 26554458 PMC4692688

[37] Turner CT, Lim D, Granville DJ. Granzyme B in skin inflammation and disease. Matrix Biol 2019;75–76:126–40. 10.1016/j.matbio.2017.12.005 29247692

[38] Hagn M, Ebel V, Sontheimer K, et al. Cd5+ B cells from individuals with systemic lupus erythematosus express Granzyme B. Eur J Immunol 2010;40:2060–9. 10.1002/eji.200940113 20394077

[39] Papp G, Gyimesi E, Szabó K, et al. Increased IL-21 expression induces Granzyme B in peripheral Cd5(+) B cells as a potential counter-regulatory effect in primary Sjögren's syndrome. Mediators Inflamm 2016;2016:4328372. 10.1155/2016/4328372 26884645 PMC4739475

[40] Cupi ML, Sarra M, Marafini I, et al. Plasma cells in the mucosa of patients with inflammatory bowel disease produce Granzyme B and possess cytotoxic activities. J Immunol 2014;192:6083–91. 10.4049/jimmunol.1302238 24835396

[41] Chesneau M, Mai HL, Danger R, et al. Efficient expansion of human Granzyme B-expressing B cells with potent regulatory properties. J Immunol 2020;205:2391–401. 10.4049/jimmunol.2000335 32948686

[42] Hagn M, Belz GT, Kallies A, et al. Activated Mouse B cells lack expression of Granzyme B. J Immunol 2012;188:3886–92. 10.4049/jimmunol.1103285 22427643

[43] Bulati M, Buffa S, Martorana A, et al. Trafficking phenotype and production of Granzyme B by double negative B cells (Igg(+)Igd(-)Cd27(-)) in the elderly. Exp Gerontol 2014;54:123–9. 10.1016/j.exger.2013.12.011 24389059

[44] Xu W, Narayanan P, Kang N, et al. Human plasma cells express Granzyme B. Eur J Immunol 2014;44:275–84. 10.1002/eji.201343711 24114594

[45] Li H, Li XL, Cao S, et al. Decreased Granzyme B(+)Cd19(+)B cells are associated with tumor progression following liver transplantation. Am J Cancer Res 2021;11:4485–99. 34659900 PMC8493381

