# Supplementary figures and images for: Impaired regulatory function of granzyme B-producing B cells against T cell inflammatory responses in lupus mice
Source: Lupus Sci Med. 2023 Jul 27;10(2):e000974. doi: 10.1136/lupus-2023-000974 (PMC10387741; doi:10.1136/lupus-2023-000974)

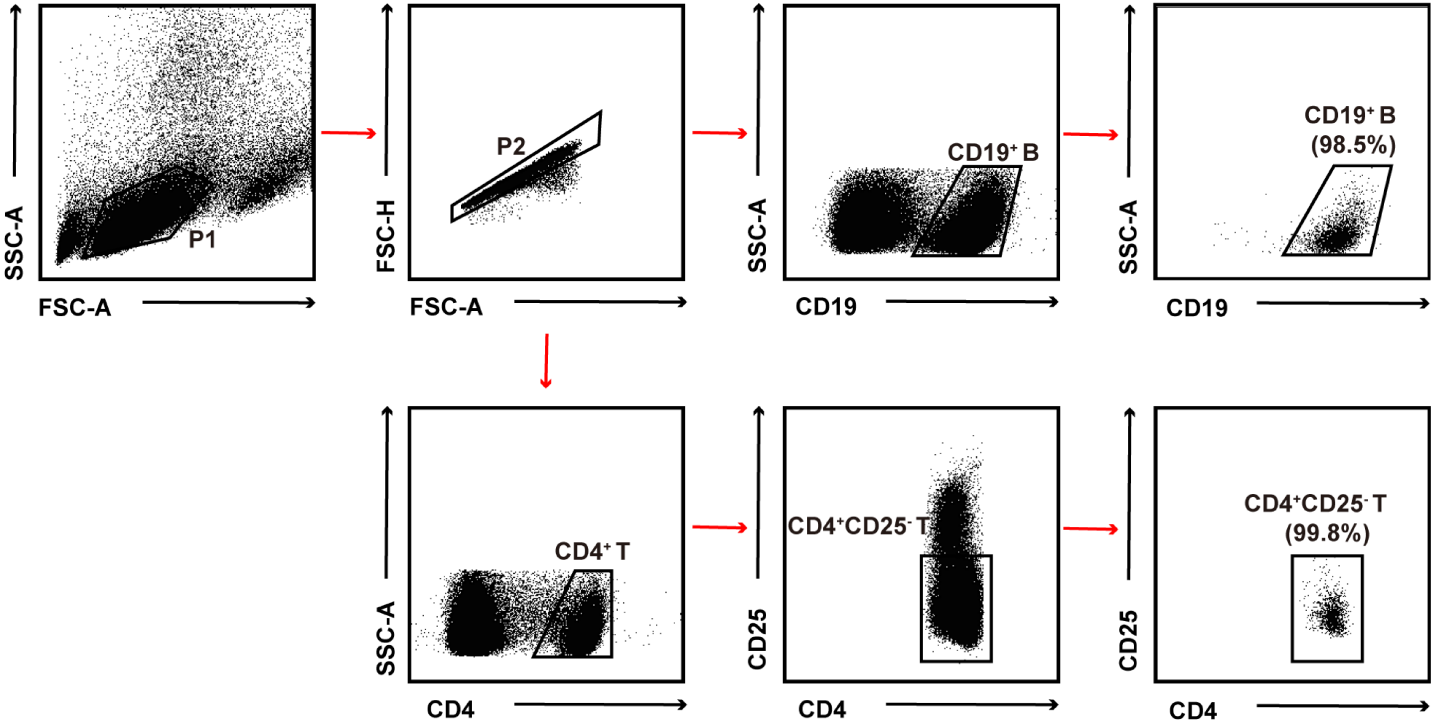

Supplement: Supplementary data [file lupus-2023-000974supp001.pdf]

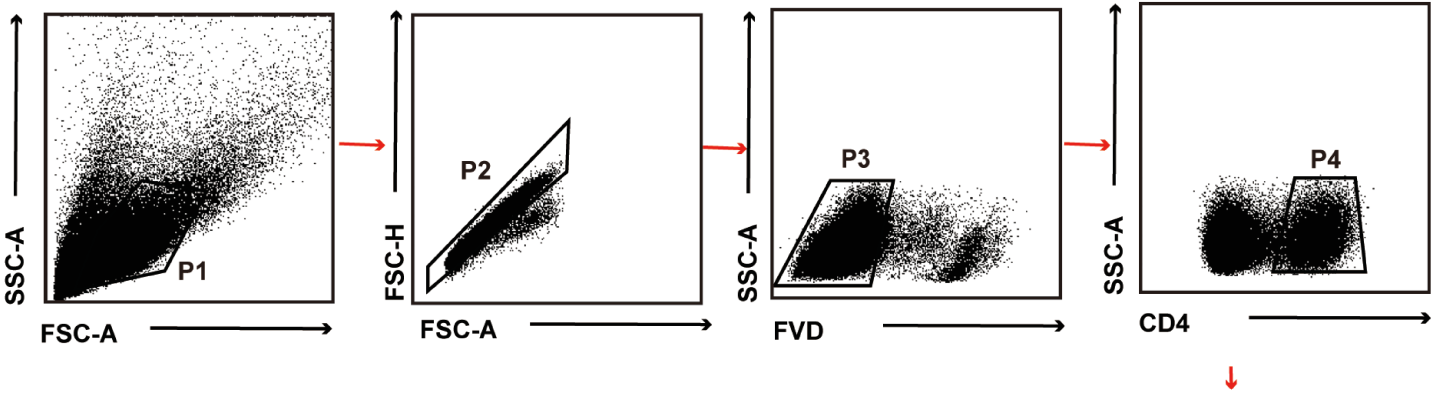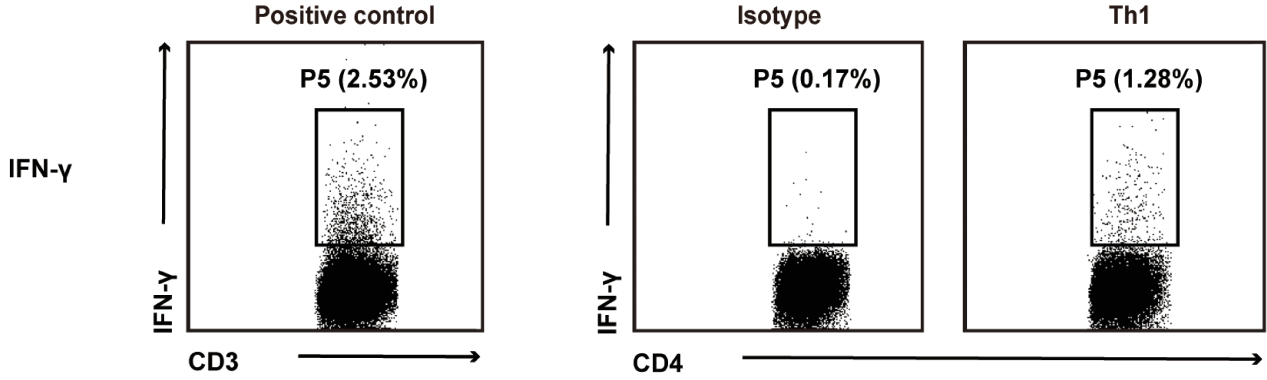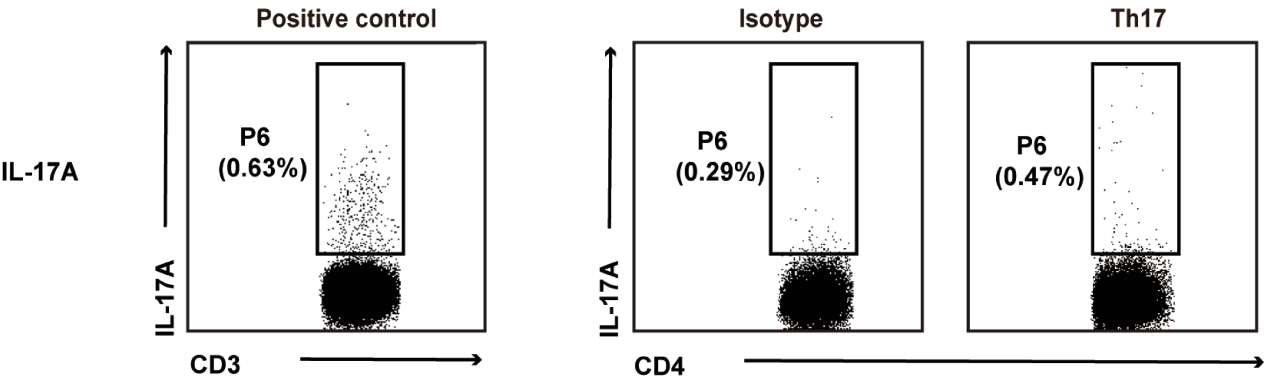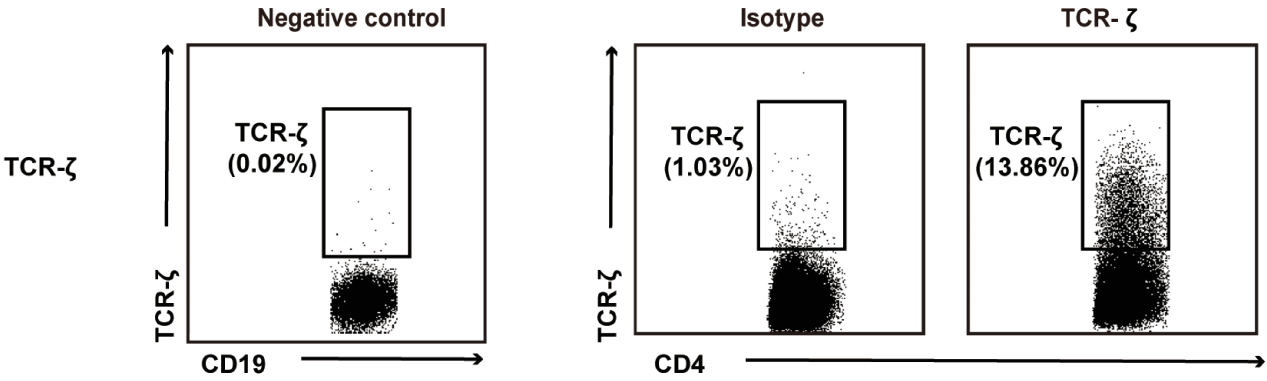

Supplement: Supplementary data [file lupus-2023-000974supp002.pdf]
